# Tuning the Photoelectrochemical Properties of Narrow Band Gap Nanoporous Anodic SnO_x_ Films by Simple Soaking in Water

**DOI:** 10.3390/ma14071777

**Published:** 2021-04-03

**Authors:** Magdalena Gurgul, Marcin Kozieł, Leszek Zaraska

**Affiliations:** Faculty of Chemistry, Jagiellonian University, Gronostajowa 2, 30-387 Krakow, Poland; gurgulm@chemia.uj.edu.pl (M.G.); marcin.koziel@uj.edu.pl (M.K.)

**Keywords:** tin oxides, anodization, nanopores, water-enabled crystallization, photoelectrochemistry

## Abstract

Nanoporous tin oxide layers obtained via anodic oxidation of metallic tin at the potential of 4 V in the alkaline electrolyte (1 M NaOH) were soaked in distilled water for various durations (from 2 h to 120 h) to verify the influence of water-enabled crystallization on the morphology, composition, and related optical and photoelectrochemical properties of such kind of anodic SnO_x_. Although water soaking generally contributes to more stoichiometric and crystalline tin oxide, it was confirmed that at the initial stages of the water-induced dissolution–redeposition process, material exhibits enhanced photoelectrochemical performance under simulated sunlight irradiation. However, long-time exposure to water results in a gradual widening of the material’s band gap, shifting of the photoelectrochemical spectra towards higher energies, and almost complete deterioration of the photoelectrochemical activity under sunlight irradiation.

## 1. Introduction

During recent years, anodically generated nanostructured tin oxide (SnO_x_) layers have received great scientific interest due to their encouraging electrochemical, optical, and semiconducting properties, which make them promising alternatives for a broad range of applications, including photoelectrochemical water splitting [1], photocatalysis [2], photovoltaics [3], energy storage systems [4,5], gas sensors [6], and others [7]. Numerous studies have indicated that these superior properties are partially a result of the nanostructured morphology of the oxide films. For this reason, the ability to control the morphology, composition, and structure of anodic metal oxides has recently gained enormous scientific attention [8]. Despite the ability to tune the morphology of anodic SnO_x_ films by modulating the conditions applied during anodic oxidation (e.g., voltage, time, temperature, and electrolyte composition) [9,10,11], the structure of the as-synthesized tin oxide materials is typically highly amorphous and independent of the electrolysis parameters [1,6,10]. Within this context, up to now, several strategies have been proposed to increase the crystallinity of the material, among which thermal annealing still seems to be the most frequently used technique [1,2,3,4,6]. It has been proven that thermal treatment of anodic tin oxide layers not only leads to partial improvement of the crystallinity but also affects the amount of Sn(II) within the material that makes a strong impact on its semiconducting properties [1,11]. However, this path cannot be fully exploited due to the low melting point of the metallic substrate on which the SnO_x_ layer resides [6,12]. Therefore, in the literature, a new strategy based on increasing the degree of crystallinity by simple storage in water has emerged. Water-enabled crystallization has been mainly studied for anodic TiO_2_ layers [13,14,15,16,17] up to 2017 when Bian and co-workers transferred these considerations to anodic tin oxide [18]; also, in our recent work [19], we verified the influence of soaking in water on the morphology, structure, and photoelectrochemical properties of anodic SnO_x_ layers obtained via potentiostatic anodization in the alkaline electrolyte at the potential of 2 V. We confirmed that exposure to water contributes to phase transformation from amorphous into the rutile type SnO_2_ by dissolution–redeposition process what significantly affects the morphology of the material. In particular, with prolonged immersion time, deterioration of the porous form of anodic layers occurred, and structure became less defective. Such changes contributed to the widening of the bandgap and dramatic decrease of the photoelectrochemical performance of the material [19].

Since we proved that anodic SnO_x_ formed at higher potential (e.g., 4 V) exhibits much wider channels and, what is extremely important, consists of much more Sn^2+^ defects resulting in significantly narrower band gaps when compared to layers grown at the potential of 2 V [1] we decided to extend our recent research. For this reason, the aim of this study was to verify whether it is possible, and if so, to what extent the simple soaking in water can be employed for tuning the morphology, crystallinity, band gap, and photoelectrochemical performance of nanoporous SnO_x_ layers grown at the higher voltage (4 V). We believe that the possibility of tuning the semiconducting properties of nanostructured tin oxide films by such an extremely simple procedure would be of great importance for various applications, especially photocatalysis and photoelectrochemical water splitting.

## 2. Materials and Methods

The Sn foil (98.8% Goodfellow, Huntingdon, England) was cut into coupons with dimensions of 1.5 cm × 1.5 cm, cleaned in acetone and ethanol, and dried. After that, the as-prepared samples were horizontally placed on the conductive plate and covered with a home-made Teflon^TM^ cell defining the working surface area. The specimens were anodized in a two-electrode setup (Pt mesh was serving as the cathode) under the constant potential difference of 4 V (Array 3646A DC power supply) in the alkaline electrolyte (1 M NaOH) for 1 h. All processes were carried out at room temperature. Afterwards, the synthesized materials were cleaned in distilled water, ethanol, and dried in the stream of warm air. Next, the non-anodized parts of the metal surface were covered with paraffin, and the samples were soaked in distilled water for diverse time durations in a range between 1 and 120 h at room temperature.

The morphology of materials was evaluated by a Field Emission Scanning Microscope (FE-SEM/EDS, Hitachi S—4700 with a Noran System 7, Hitachi, Tokyo, Japan), and morphological features of tin oxide were estimated from the SEM images by image processor WSxM v. 12.0. XRD patterns of all samples were collected using the PANalytical X’Pert PRO MPD diffractometer in Bragg–Brentano geometry (Malvern, UK), using a copper X-ray source over the 2θ range of 25–70°. Powder patterns were cross-referenced with the PDF-4+ database [20] using X’Pert HighScore commercial software [21].

UV–vis reflectance spectra were recorded using a Lambda 750S spectrophotometer (Perkin-Elmer, Waltham, MA, USA) equipped with an integrating sphere module. Diffuse reflectance spectra (DRS) of samples were collected in the range of 250–800 nm with a step size of 2 nm. Spectralon^®^ SRS-99-010 was used as a reference. The data were processed using Perkin Elmer UV WinLab Data Processor and Viewer. 

Photoelectrochemical (PEC) measurements were carried out using the photoelectric spectrometer combined with a potentiostat (Instytut Fotonowy, Krakow, Poland) equipped with the 150 V Xe arc lamp. The measurements were performed in a conventional three-electrode system with SnO_x_ layers serving as working electrodes and Pt wire and saturated calomel electrode (SCE) as a counter and reference electrodes, respectively. The photocurrents were recorded in a borate buffer solution (pH ~7.4) at the potential of 1 V vs. SCE under sequential illumination with monochromatic light in a range between 250 and 800 nm. Moreover, chronoamperometric measurements were also performed under sequential illumination with AM 1.5 G standard sunlight using a xenon illuminator 150 W (Instytut Fotonowy) combined with PalmSens4 (PalmSens BV, Houten, The Netherlands) potentiostat.

## 3. Results

**Figure 1:**
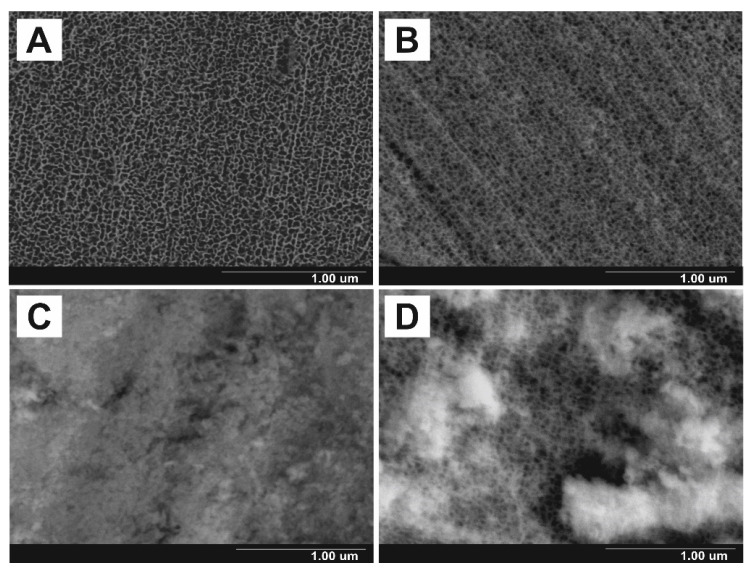
Field Emission Scanning Microscope (FE-SEM) images of nanoporous tin oxide layers as-grown at the potential of 4 V for 1 h (**A**) and soaked in distilled water for 2 h (**B**), 72 h (**C**), and 120 h (**D**).

The FE-SEM images of nanoporous SnO_x_ layers grown at the potential of 4 V for 1 h and soaked for diverse time periods are shown in Figure 1. As can be seen, anodically generated tin oxide layers exhibit highly porous morphology with well-defined and randomly distributed channels. For as-received material (Figure 1A), the estimated pore diameter was ~35 nm which is consistent with our previous works [1,12]. It should be emphasized that channels are significantly larger when compared to those observed within the layers grown at 2 V (<20 nm), as we proved in our recent paper [19].

In comparison to the as-received material, when the samples were immersed in distilled water, it caused significant morphological changes of anodic layers. As shown in Figure 1B, after 2 h of soaking, the surface of porous film becomes more roughened what is accompanied by a noticeable decrease in pore diameter to ca. 30 nm. After long-term exposure to water, the pores are almost completely clogged with a secondary deposit having highly granular morphology (Figure 1C,D). As we discussed in detail previously [19], these changes are a result of dissolution–redeposition processes. However, as can be seen in Figure 1D, even after 120 h of soaking, the porous morphology is still present within the internal part of the layer. Contrary to this, we recently showed that after soaking for the same time, anodic SnO_x_ films grown at 2 V were found to be almost completely compact [19]. This discrepancy could be attributed to much wider channels within the layers grown at higher potentials (see above). Nevertheless, a gradual narrowing of pores was observed in the present case, and the determined values of pore diameter are 26 nm and 19 nm for layers immersed in water for 72 h and 120 h, respectively.

**Figure 2:**
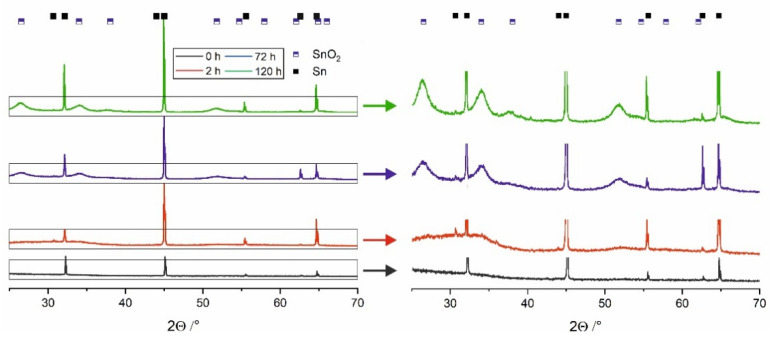
XRD pattern of samples anodized at 4 V for 1 h and soaked in the water for 2 h, 72 h, and 120 h.

As already mentioned, directly after the anodization process, oxide film layers are amorphous and independent of the applied voltage or electrolyte type [1,7,19]. On the contrary, XRD patterns obtained for the materials stored in water (Figure 2) exhibit noticeable maxima located at 26.6°, 34.1°, 37.6°, 51.8°, and 66.1° that can be attributed to the cassiterite SnO_2_ phase. As expected, these maxima become more intense and narrower with increasing the immersing time what denotes for progressive phase transformation of SnO_x_ into more stoichiometric and crystalline SnO_2_. The process of water-enabled crystallization of anodic SnO_x_ was discussed in detail in our recent paper [19] and by Bian et al. [18].

**Figure 3:**
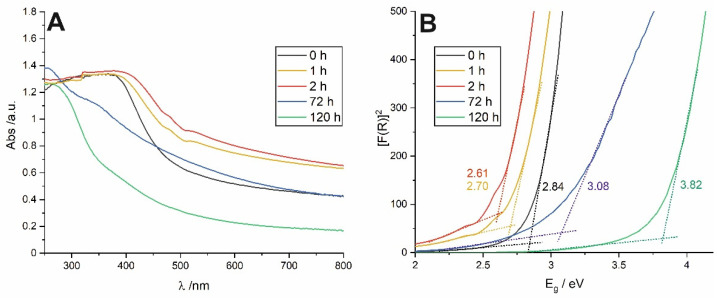
UV–VIS reflectance spectra of nanostructured SnO_x_ obtained after anodic oxidation at 4 V and soaked in the water for different durations (**A**) with the corresponding [F(R) hν]^2^ vs. hν plots obtained for all studied samples (**B**).

Anodic tin oxide layers synthesized at the potential of 4 V exhibit yellow-brown color, much darker than those obtained at lower potential (2 V) [1,13]. The reason for that is their highly defective nature being responsible for the significant absorption in the visible range [11,13]. After 2 h of soaking, the color of the anodic films becomes deeper, while prolonged exposure to water results in a gradual color fading. This relation was confirmed by UV–VIS reflectance spectra. It is clear from Figure 3A that the as-received layer exhibit much greater absorption in the visible range (black line), and its absorption edge is located at longer wavelengths (lower energies) when compared to the material soaked for 120 h (green line).

According to the procedure described in our previous works [13,19], optical band gap values (E_g_) were also determined for all studied samples. Briefly, at first, Kubelka-Munk (F(R)) functions were calculated from UV–VIS spectra, and then [F(R) hν]^2^ vs. hν plots (Tauc plots) were constructed (see Figure 3B). E_g_ values were found by extrapolation of the straight regions to the baseline. A relatively narrow band gap observed for the as-anodized sample (~2.8 eV) is in line with our previous findings [1,13], and the value corresponds to the yellow-orange color of the material. Surprisingly, contrary to our recent findings for anodic films formed at 2 V [19] after 1 and 2 h of soaking, a noticeable and gradual narrowing of E_g_ (to ca. 2.6 eV for sample soaked for 2 h) was observed.

**Figure 4:**
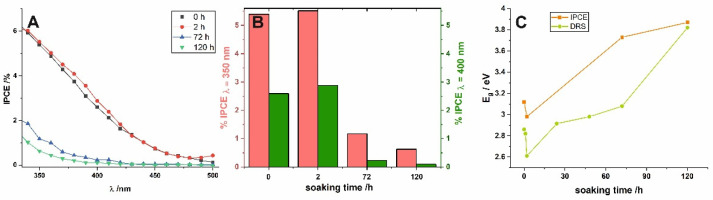
Incident photon to current efficiency (IPCE) spectra of as-anodized layers and immersed in water for various durations (**A**), calculated IPCE at 350 nm and 400 nm at various times (**B**), the relation between diffuse reflectance spectra (DRS) and IPCE band gaps calculated for all layers (**C**).

This behavior can be explained as follows. As we discussed in detail in our recent works [1,13], anodic SnO_x_ films formed at the potential of 4 V are much more defective than those grown at 2 V that corresponds to a much lower E_g_ value. Despite the fact that it is known that spontaneous oxidation of SnO to SnO_2_ in water can occur [19], a short-term soaking is not enough to achieve noticeable changes in the number of defects (for instance, for SnO_x_ layers formed at the potential of 2 V no noticeable changes in E_g_ after 2 exposure to water was observed—see ref. [19]). However, in the present case, the higher Sn^2+^ content within the self-doped SnO_2_ can lead firstly to the local crystallization Sn^2+^-rich domains in the structure (similarly to the phenomena observed during thermal treatment of this material at relatively low temperatures—see ref. [13]) or even to the local formation of intermediate oxides (e.g., Sn_3_O_4_) resulting in a gradual narrowing of E_g_. Further long-term soaking results in a progressive dissolution and redeposition of less defective and more stoichiometric SnO_2_ rutile phase what is accompanied by a band gap widening (the trend was also confirmed by checking the samples soaked for 24 and 48 h, as presented in Figure 4C. Similar behavior was also observed for layers grown at 2 V [19]. It should be also mentioned that significantly different morphology of anodic films formed at 4 V compared to those generated at 2 V (especially much greater thickness and wider nanochannels) can be also the reason for noticeable different E_g_ changes during water-enabled crystallization. However, a deeper understanding of this phenomenon requires some additional studies, which are planned in the near future.

Photoelectrochemical (PEC) performance of short-term and long-term soaked samples was also investigated to verify the influence of water-enabled crystallization on the PEC properties of nanoporous anodic SnO_x_. Incident photon to current efficiency (IPCE) values were calculated for all characterized photoanodes using the following equation:

$$IPCE=1240\frac{J_{p}\left(\lambda \right)}{\lambda \;P\left(\lambda \right)}$$

where *J_p_* (*λ*)—photocurrent density at the particular wavelength, *λ*—wavelength, *P*(*λ*)—power density at the particular wavelength, and the resulting IPCE spectra are collected in Figure 4. Firstly, a noticeable photoresponse of the as-received sample in the visible range should be highlighted (see the black line in Figure 4A). Comparing IPCE spectra obtained for as-received anodic film and the sample soaked for longer durations (Figure 4A), it is clear that the long exposure to water causes a significant shift of the photoresponse to higher energy values. This is strongly in line with the results observed previously for anodic layers synthesized at the lower potential (2 V) [19] and perfectly fits the gradual widening of E_g_ observed from UV–VIS spectra (see above). To confirm this, (IPCE hν)^2^ vs. hν plots were constructed (not shown here) and the E_g_ values were also estimated by extrapolation of the linear parts to the energy axis. As can be seen in Figure 4C, the dependence between E_g_ and immersion time was found to be almost identical and independent of the method used for band gap determination.

A detailed comparison of IPCE values (Figure 4B), reveals that the best photoelectrochemical activity was observed for samples soaked for 2 h. This can be attributed to the fact that such short exposure to water did not cause a significant change in the degree of self-doping, i.e., the number of charge carriers is still very high, while the crystallinity of the material is slightly improved. Finally, the more compact surface morphology of the anodic films caused by the dissolution–redeposition process may be, in addition to reducing the number of defects and formation of more crystalline and stoichiometric SnO_2_ [19], the reason for the worse photoelectrochemical performance of long-term soaked photoanodes. However, further and more detailed studies are mandatory to understand the described phenomena completely.

**Figure 5:**
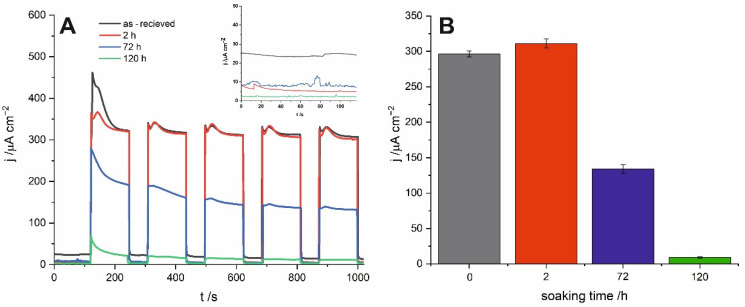
Chronoamperometric curves recorded under sequential illumination for anodic tin oxide layers as-obtained and soaked in distilled water (**A**), photocurrent values after subtracting dark currents recorded for the materials (**B**).

Chronoamperometric curves recorded for all studied samples under sequential illumination with simulated sunlight are presented in Figure 5A. Similarly to the already delineated trend, material soaked in water for 2 h exhibits the highest and the most stable photocurrents. Moreover, as shown in the inset of Figure 5A, a short time exposure to water results in a significant decrease in the dark currents and, in consequence, increases the stability of the SnO_x_ photoanode. For the material immersed for 120 h, photocurrents are almost negligible under solar irradiation what is strongly in line with the blue shift of the photocurrent spectra and the band gap widening described above.

Finally, it is worth mentioning that a significant worsening of PEC activity manifested in lower photocurrents and blue shift of the photocurrent spectra was also observed for anodic tin oxide layers after annealing in air at 400 °C. Such kind of thermal treatment resulted in a similar increase in crystallinity and band gap widening like in the case of water-enabled crystallization. The main difference was that nanoporous morphology was still maintained after thermal annealing, while long-term soaking in water resulted in clogging of the porous surface. However, comparing the photoelectrochemical activity of short-term soaked samples with those thermally treated at relatively low temperature (200 °C), it can be concluded that despite noticeable improvement in the photoelectrochemical performance of anodic SnO_x_ formed at 4 V after short exposure to water, a thermal treatment still remains the more promising strategy to enhance the efficiency of anodically generated tin oxide photoanodes.

## 4. Conclusions

In summary, we characterized the influence of water-enabled crystallization on the anodic tin oxide films obtained in the alkaline electrolyte at the potential of 4 V. Similarly to our recent research, we verified the changes in morphology induced by the immersion and the related changes in the properties of the nanostructured oxide. We have proven that water immersion causes a spontaneous phase transformation of amorphous tin oxide to the rutile-type SnO_2_. Contrary to the trend observed for layers obtained at lower potentials in our recent work, in the present case, material soaked for a short time exhibited improved photoelectrochemical activity, probably due to the slightly improved crystallinity of the material together with its highly defective nature. Although the presented results still require in-depth analysis and additional research, it is undoubted that the post-treatment path of nanostructured SnO_x_ after the anodization process possesses a huge impact on its structure and morphology and, thus, also on its properties.

## Data Availability

Data sharing not applicable.
